# Case Report: An atypical case of ARPKD highlights the utility and challenges of implementing genetic testing in cystic kidney disease

**DOI:** 10.3389/fped.2025.1677417

**Published:** 2025-11-03

**Authors:** Jonathan Marquez, Lauren M. Hawkins, Anita E. Beck, Katrina M. Dipple, Ian A. Glass, Alexandra C. Keefe, Elizabeth D. Nguyen

**Affiliations:** ^1^ Department of Pediatrics, Division of Genetic Medicine, University of Washington and Seattle Children’s Hospital, Seattle, WA, United States; ^2^ Center for Developmental Biology and Regenerative Medicine, Seattle Children’s Research Institute, Seattle, WA, United States; ^3^ Department of Pediatrics, Division of Nephrology, University of Washington and Seattle Children’s Hospital, Seattle, WA, United States; ^4^ Brotman Baty Institute for Precision Medicine, University of Washington, Seattle, WA, United States; ^5^ Center for Clinical and Translational Research, Seattle Children’s Research Institute, Seattle, WA, United States; ^6^ Center for Integrative Brain Research, Seattle Children’s Research Institute, Seattle, WA, United States

**Keywords:** autosomal recessive polycystic kidney disease, exome sequencing, genetics, inherited kidney disease, cystic liver disease

## Abstract

**Background:**

Biallelic pathogenic variants in *PKHD1* cause a highly heterogenous disease, predominantly involving the kidneys and liver. Although the correlation between genotype and phenotype remains unclear, many variants in this gene have been described.

**Case:**

In this study, we describe a case of suspected autosomal recessive polycystic kidney disease (ARPKD) due to a novel variant in *PKHD1*. The patient in this instance presented with a novel *PKHD1* variant (c.2713C>A; p.Gln905Lys) in *trans* with a previously described pathogenic variant (c.7994T>C; p.Leu2665Pro).

**Conclusions:**

The *PKHD1* variant c.2713C>A; p.Gln905Lys may contribute to an ARPKD phenotype with a delayed juvenile onset.

## Introduction

Autosomal recessive polycystic kidney disease (ARPKD) is a rare condition that arises due to biallelic pathogenic variants in a small subset of genes, the most common of which is *PKHD1*. *PKHD1* encodes the fibrocystin protein that participates in the development of kidney and liver tubular structures. Fibrocystin is understood to function through a variety of proposed roles that include acting as a transmembrane ciliary protein and signaling the release of C-terminal fragments (1–3). While the genetic etiology of ARPKD has long been appreciated, reports of this condition have predominantly focused on severe presentations during the perinatal period (4–6). As sequencing-based genetic testing has become more prevalent in the evaluation of individuals with kidney disease, a broader phenotypic spectrum of ARPKD, which has a later onset, has become apparent (7, 8). Indeed, juvenile- and adult-onset ARPKD has been reported and appears to follow a distinct clinical course with less severe kidney disease and often a predominance of liver-related complications (9, 10). Yet, it remains unclear how different genetic variants may contribute to varying ARPKD phenotypes. In this study, we present the case of an individual with a clinical diagnosis of ARPKD, which is thought to have arisen due to compound heterozygous variants in *PKHD1* and includes a novel variant in this gene detected through exome sequencing (ES).

## Case report

**Figure 1:**
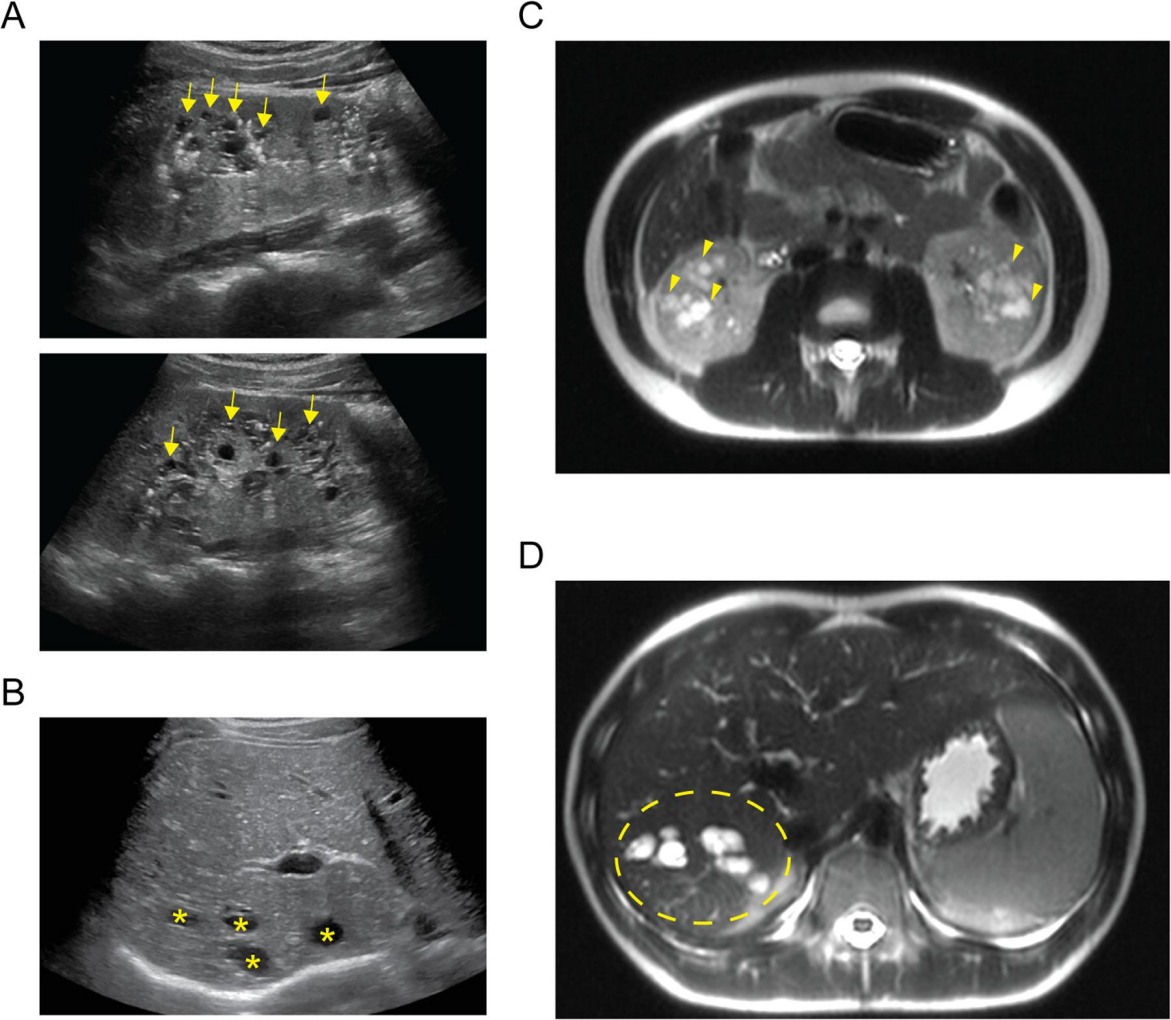
Imaging of cystic organ changes. **(A)** Sagittal ultrasound images of right (upper) and left (lower) kidneys demonstrating diffuse cystic changes predominantly affecting the medullary parenchyma (examples indicated with yellow arrows). **(B)** Transverse ultrasound image of the liver demonstrating cystic spaces of varying size affecting the hepatic parenchyma (examples indicated with yellow asterisks). **(C)** Axial T2-weighted MRI cross section demonstrating extensive kidney cystic changes bilaterally (examples indicated with yellow arrowheads). **(D)** Axial T2-weighted MRI cross section demonstrating extensive cystic changes within the right hepatic lobe (affected area within area demarcated by yellow dashed ellipse).

A 14-year-old XY boy initially presented to the nephrology clinic following an abdominal ultrasound that found multiple cortical kidney cysts and multiple hepatic cysts (Figures 1A,B). On initial laboratory evaluation, he was found to have abnormalities consistent with chronic kidney disease (CKD). He had a serum creatinine level of 1.15 mg/dL, albumin of 4.7 g/dL, blood urea nitrogen of 26 mg/dL, potassium of 4.3 mmol/L, bicarbonate of 21 mmol/L, calcium of 9.9 mmol/L, and 1+ protein in his urine. He underwent magnetic resonance imaging (MRI) of the abdomen to further delineate the cystic structures (Figures 1C,D). His liver and kidney cysts had a maximal volume of 14,137 and 179 mm^3^, respectively. His history was significant with a previously identified small unspecified number of hepatic cysts found incidentally during ultrasound evaluation in the setting of a prior gastrointestinal illness at 8 months of age. His kidneys were not cystic during the prior imaging, and he had no dietary history that would contribute to decreased kidney function. Similarly, he had no history of severe or frequent infections that would increase his risk for decreased kidney function. He was not taking any routine medication and had no history of exposure to known nephrotoxic agents.

Due to the presence of multi-organ cysts along with evidence of proteinuria, he was referred to the medical genetics department for further evaluation. His results did not show evidence of dysmorphic craniofacial features or of a well-described syndrome. His development had progressed typically following an uncomplicated pregnancy. His family history included notable episodes of hepatic cysts and hepatic hemangiomas in his father, which had been attributed to a history of hepatitis B infection. As a genetic cause of cystic hepatorenal disease was suspected, diagnostic testing in the form of clinical ES was performed, with concurrent copy number variant analysis. This test was carried out by a clinical laboratory improvement amendments (CLIA)–certified laboratory and included exon capture and sequencing of coding exons plus ±10 bp of flanking non-coding DNA for each exon. Exome sequencing when carried out in this manner, with analysis that considers the phenotype, can detect pathogenic variants in many of the potential causes of cystic kidney and/or cystic liver disease.

**Figure 2:**
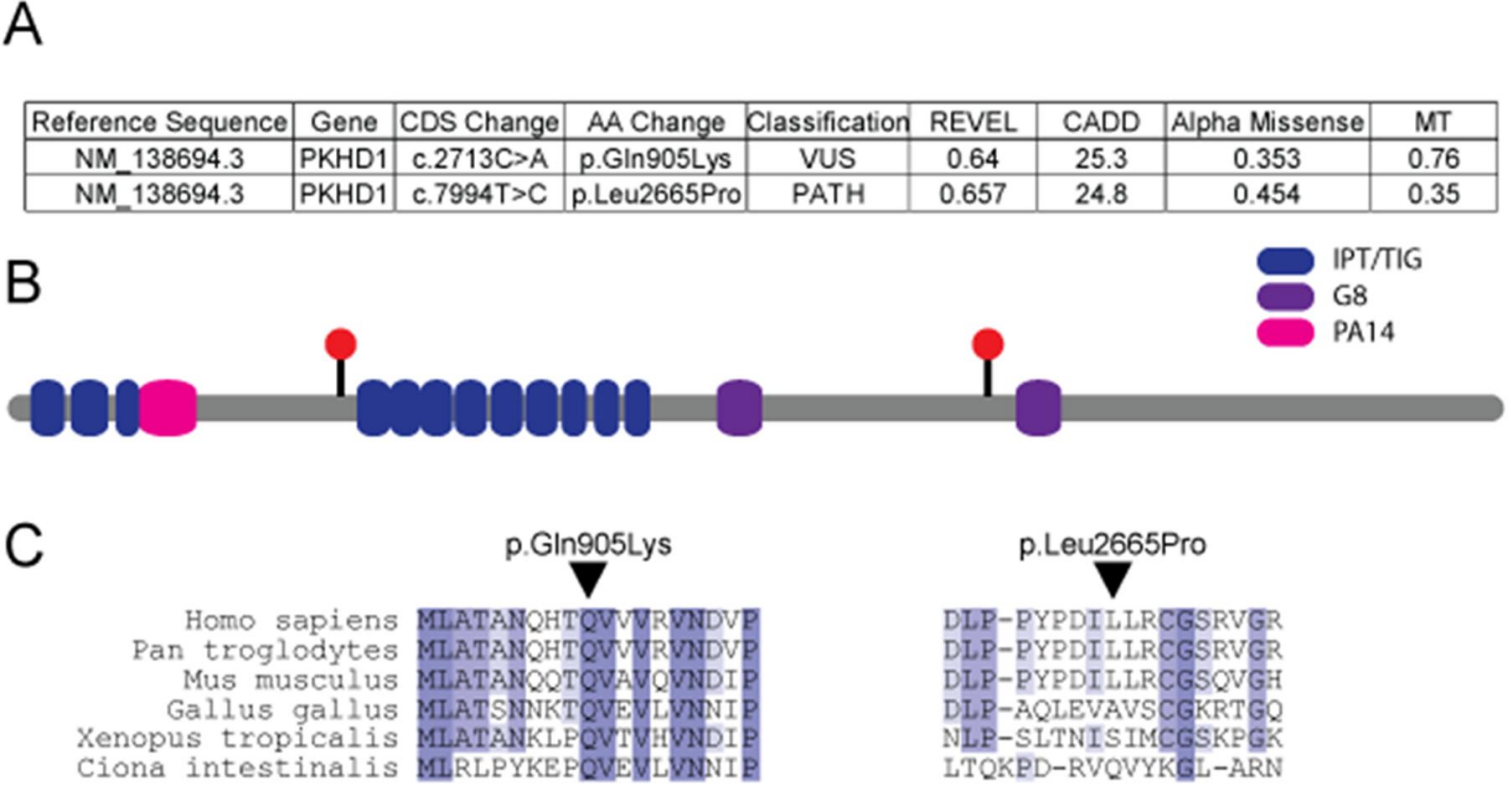
Assessment of identified genetic changes in *PKHD1*. **(A)** Table detailing *PKHD1* genetic variants observed in the reported individual and *in silico* predictions of the effects of these variants. Individual metrics were accessed directly from each source. **(B)** Location of the two missense variants in the context of the protein domains of fibrocystin displayed as red circles. **(C)** Conservation through phylogeny for the two *PKHD1* variants. AA, amino acid; CADD, combined annotation-dependent depletion; CDS, coding sequence; G8, eight conserved glycines; IPT/TIG, immunoglobulin-like fold, plexins, transcription factors/transcription factor immune globin; MT, mutation taster; PA14, protective antigen 14; PATH, pathogenic; REVEL, rare exome variant ensemble learner; VUS, variant of uncertain significance.

Analysis of sequencing results was completed with particular emphasis on those genes known to be associated with cystic kidney and/or liver disease. This testing identified compound heterozygous variants in *PKHD1* (Figure 2A). One of these was maternally inherited (NM_138694.3 *PKHD1* c.7994T>C; p.Leu2665Pro) and classified as pathogenic. The other variant was paternally inherited (NM_138694.3 *PKHD1* c.2713C>A; p.Gln905Lys) and classified as a variant of uncertain significance (VUS). The classifications were completed by the clinical laboratory in accordance with American College of Medical Genetics (ACMG) guidelines (11). These variants were deposited in the ClinVar database under accession numbers SCV005339882.1 and SCV005363611.1. While the pathogenicity of the detected VUS remains unclear, given the patient’s clinical course and imaging findings, *PKHD1*-related autosomal recessive polycystic kidney disease (*PKHD1*-ARPKD) was deemed a likely diagnosis. Indeed, another variant at fibrocystin amino acid 905 has been classified as pathogenic. However, this is a truncating variant (Gln905Ter) previously identified in individuals with severe neonatal ARPKD (12, 13). The p.Gln905Lys missense variant is extremely rare in the population, and multiple *in silico* prediction tools further support the deleterious nature of the missense change for this VUS (Figure 2A). While some variants implicated in ARPKD, such as splice site variants (14), are amenable to testing to determine possible pathogenicity, missense variants such as the one we describe are not easily tested for effect on function. The amino acid position lies near the immunoglobulin-like fold, plexins, transcription factors/transcription factor immune globin (IPT/TIG) domains of fibrocystin that have an immunoglobulin-like fold where numerous missense variants putatively implicated in ARPKD phenotypes are found (Figure 2B). In addition, this amino acid position is evolutionarily conserved to the level of invertebrates (Figure 2C).

The patient’s treatment proceeded in a manner consistent with the surveillance and interventions recommended for ARPKD. His blood pressure was closely monitored and was predominantly within reference ranges for age, and his 24-h ambulatory blood pressure monitoring was also within normal limits. His treatment included evaluation by hepatologists, along with ongoing care in both nephrology and genetics clinics. His kidney function remained relatively stable with modest improvement in proteinuria after initiation of an angiotensin-converting enzyme inhibitor, which he tolerated without a significant rise in creatinine. His hepatic function was thus far within reference laboratory ranges. However, this represented a limited window for clinical observation, and the long-term kidney and hepatic disease course for this patient remains uncertain.

## Discussion

We identified biallelic *PKHD1* variants in an individual presenting with kidney dysfunction and cystic kidney and liver morphology in adolescence. This included one VUS in *trans* with a pathogenic variant. This individual's family history was suggestive of an autosomal dominant disorder based on his father's cystic liver finding, which nevertheless rendered this less straightforward due to the father's hepatitis history. Indeed, though autosomal dominant polycystic kidney disease (ADPKD) is much more common (15, 16), it follows a different disease course compared to ARPKD (17). This individual's imaging findings of medullary predominance of small kidney cysts and biliary cystic changes are less consistent with ADPKD presenting in childhood and more consistent with what has been described in ARPKD with later childhood and adult onset (18). While we considered whether his findings could be caused by monoallelic *PKHD1* variants, which have been previously described (19–21), this apparently distinct disorder typically includes increased medullary echogenicity of the kidney and multiple small liver cysts, which are inconsistent with the patient's radiological findings. We also considered whether another genetic cause could have remained undetected by the clinical exome sequencing approach used. Our knowledge of all potential genes in which variants might lead to cystic kidney disease is likely incomplete, and this may serve as a limitation to clinically identifying relevant genetic variants. However, many variants that give rise to cystic kidney and liver disease often also occur alongside other clinical manifestations. For instance, variants in genes such as *ALG8* or *ALG9* often result in a complex neurodevelopmental disorder of glycosylation (22, 23). As additional manifestations were not observed in this case, many other genetic causes of cystic tissue morphology were deemed less likely.

An individualized approach to treatment is beneficial in children and adolescents with ARPKD, and interventions should be tailored to the evidence of CKD progression, persistent hypertension, and/or complications from hepatic involvement, such as portal hypertension or cholangitis (24). Disease surveillance to detect these indications for further intervention should be multidisciplinary and tailored to disease severity. Appropriate measures typically include regular blood pressure monitoring, laboratory evaluations of kidney function and electrolytes, hepatic surveillance through both laboratory evaluations and imaging of the liver, and monitoring for psychosocial complications of disease (25). Particular emphasis and increased frequency of evaluation may also be useful during puberty, as CKD has been described to accelerate during this physiological life stage, which could also be the case in the context of CKD due to ARPKD (26).

Even among large cohorts of children and adolescents diagnosed with ADPKD from high- and middle-income countries, only approximately half individuals or fewer have undergone genetic testing (27). The genetic etiology of cystogenesis in individuals with a clinical diagnosis of ADPKD may well range beyond variants in *PKD1* and *PKD2*. ARPKD is more frequently considered in the evaluation of perinatal hepatic and kidney disease, given the many reports of early-presenting ARPKD in the literature (28, 29). Indeed, the patient has a personal and family history that could have led to the clinical diagnosis of ADPKD, albeit with an earlier presentation than typical. However, genetic testing, despite a VUS in *trans* with a pathogenic variant in *PKHD1*, facilitated a more tailored approach to his treatment and likely a more accurate prognosis. Recent efforts have attempted to delineate the clinical course of ARPKD in young adults (30). Based on these data, it appears that individuals with ARPKD have a greater likelihood of stable native kidney function into adulthood despite varying levels of CKD compared to other diagnosed causes of cystic kidney disease, such as ADPKD. In addition, such individuals frequently develop fibrotic liver disease and resultant portal hypertension, necessitating multi-drug blood pressure management later in life. Despite these insights, even in larger cohorts of individuals with ARPKD diagnoses, fewer than half of the reported individuals have undergone genetic testing (30). This limits our ability to identify genotype–phenotype correlations that could further improve counseling related to ARPKD diagnoses.

Therefore, while genetic testing in the setting of cystic kidney disease certainly necessitates comprehensive genetic counseling tailored to each individual, as has been suggested by others (31), we recommend that the benefits of testing to achieve tailored care are considerable and should be more widely available. Nevertheless, we also acknowledge the challenges of uncertain results, such as seen in this case. While VUSs are often a component of genetic testing results, it seems likely that these may be an even more prevalent component of *PKHD1*-ARPKD-related testing for older children and adults, since missense variants do seem to yield a milder and later-onset phenotype that has not been as well captured in the literature thus far (28). Therefore, interpretation of testing may well rely on careful consideration of predicted molecular effects of a variant, taken along with the clinical presentation in the case of uncertain results, as illustrated here.

## Conclusion

This case demonstrates a rare phenotype of suspected *PKHD1*-ARPKD manifesting in adolescence. We describe a novel variant in *PKHD1* that likely contributes to a later-onset liver-predominant phenotype with cystic kidney changes. Genetic testing is an important consideration for individuals with cystic kidney disease, as a suggestive or conclusive molecular diagnosis can greatly influence management.

## Data Availability

The original contributions presented in the study are included in the article/Supplementary Material, further inquiries can be directed to the corresponding authors.

## References

[1] Ward CJ Hogan MC Rossetti S Walker D Sneddon T Wang X The gene mutated in autosomal recessive polycystic kidney disease encodes a large, receptor-like protein. Nat Genet. (2002) 30:259–69. 10.1038/ng833 11919560

[2] Walker RV Yao Q Xu H Maranto A Swaney KF Ramachandran S Fibrocystin/polyductin releases a C-terminal fragment that translocates into mitochondria and suppresses cystogenesis. Nat Commun. (2023) 14:6513. 10.1038/s41467-023-42196-4 37845212 PMC10579373

[3] Onuchic LF Furu L Nagasawa Y Hou X Eggermann T Ren Z PKHD1, the polycystic kidney and hepatic disease 1 gene, encodes a novel large protein containing multiple immunoglobulin-like plexin-transcription–factor domains and parallel beta-helix 1 repeats. Am J Hum Genet. (2002) 70:1305–17. 10.1086/340448 11898128 PMC447605

[4] Beaunoyer M Snehal M Li L Concepcion W Salvatierra O Jr Sarwal M. Optimizing outcomes for neonatal ARPKD. Pediatr Transplant. (2007) 11:267–71. 10.1111/j.1399-3046.2006.00644.x 17430481

[5] Adeva M El-Youssef M Rossetti S Kamath PS Kubly V Consugar MB Clinical and molecular characterization defines a broadened spectrum of autosomal recessive polycystic kidney disease (ARPKD). Medicine (Baltimore). (2006) 85:1. 10.1097/01.md.0000200165.90373.9a 16523049

[6] Bean SA Bednarek FJ Primack WA. Aggressive respiratory support and unilateral nephrectomy for infants with severe perinatal autosomal recessive polycystic kidney disease. J Pediatr. (1995) 127:311–3. 10.1016/S0022-3476(95)70318-7 7636663

[7] Zhang X Wu J Zhou J Liang J Han Y Qi Y Pathogenic relationship between phenotypes of ARPKD and novel compound heterozygous mutations of PKHD1. Front Genet. (2024) 15:1429336. 10.3389/fgene.2024.1429336 39015774 PMC11250243

[8] Das A Mead P Sayer JA. Adult presentations of variable kidney and liver phenotypes secondary to biallelic PKHD1 pathogenic variants. J Rare Dis. (2023) 2:1. 10.1007/s44162-022-00002-7

[9] Blyth H Ockenden BG. Polycystic disease of kidney and liver presenting in childhood. J Med Genet. (1971) 8:257–84. 10.1136/jmg.8.3.257 5097134 PMC1469189

[10] Fonck C Chauveau D Gagnadoux M Pirson Y Grünfeld J. Autosomal recessive polycystic kidney disease in adulthood. Nephrol Dial Transplant. (2001) 16:1648–52. 10.1093/ndt/16.8.1648 11477168

[11] Richards S Aziz N Bale S Bick D Das S Gastier-Foster J Standards and guidelines for the interpretation of sequence variants: a joint consensus recommendation of the American College of Medical Genetics and Genomics and the Association for Molecular Pathology. Genet Med. (2015) 17:405–24. 10.1038/gim.2015.30 25741868 PMC4544753

[12] Ishiko S Morisada N Kondo A Nagai S Aoto Y Okada E Clinical features of autosomal recessive polycystic kidney disease in the Japanese population and analysis of splicing in PKHD1 gene for determination of phenotypes. Clin Exp Nephrol. (2022) 26:140–53. 10.1007/s10157-021-02135-3 34536170 PMC8770369

[13] Obeidova L Seeman T Elisakova V Reiterova J Puchmajerova A Stekrova J. Molecular genetic analysis of PKHD1 by next-generation sequencing in Czech families with autosomal recessive polycystic kidney disease. BMC Med Genet. (2015) 16:116. 10.1186/s12881-015-0261-3 26695994 PMC4689053

[14] Molinari E Srivastava S Dewhurst RM Sayer JA. Use of patient derived urine renal epithelial cells to confirm pathogenicity of PKHD1 alleles. BMC Nephrol. (2020) 21:435. 10.1186/s12882-020-02094-z 33059616 PMC7559414

[15] Aung TT Bhandari SK Chen Q Malik FT Willey CJ Reynolds K Jacobsen SJ Sim JJ. Autosomal dominant polycystic kidney disease prevalence among a racially diverse United States population, 2002 through 2018. Kidney360 (2021) 2:2010. 10.34067/KID.0004522021 35419536 PMC8986058

[16] Willey CJ Blais JD Hall AK Krasa HB Makin AJ Czerwiec FS. Prevalence of autosomal dominant polycystic kidney disease in the European Union. Nephrol Dial Transplant. (2017) 32:1356–63. 10.1093/ndt/gfw240 27325254 PMC5837385

[17] Halawi AA Burgmaier K Buescher AK Dursun I Erger F Galiano M Clinical characteristics and courses of patients with autosomal recessive polycystic kidney disease-mimicking phenocopies. Kidney Int Rep. (2023) 8:1449–54. 10.1016/j.ekir.2023.04.006 37441483 PMC10334384

[18] Turkbey B Ocak I Daryanani K Font-Montgomery E Lukose L Bryant J Autosomal recessive polycystic kidney disease and congenital hepatic fibrosis (ARPKD/CHF). Pediatr Radiol. (2009) 39:100–11. 10.1007/s00247-008-1064-x 19089418 PMC2918426

[19] Gunay-Aygun M Turkbey BI Bryant J Daryanani KT Gerstein MT Piwnica-Worms K Hepatorenal findings in obligate heterozygotes for autosomal recessive polycystic kidney disease. Mol Genet Metab. (2011) 104:677–81. 10.1016/j.ymgme.2011.09.001 21945273 PMC3224207

[20] de Fallois J Schönauer R Münch J Nagel M Popp B Halbritter J. Challenging disease ontology by instances of atypical PKHD1 and PKD1 genetics. Front Genet. (2021) 12:682565. 10.3389/fgene.2021.682565 34249099 PMC8267867

[21] Van Buren JD Neuman JT Sidlow R. Predominant liver cystic disease in a new heterozygotic PKHD1 variant: a case report. Am J Case Rep. (2023) 24:1–5. 10.12659/AJCR.938507 PMC9883601 36691356

[22] Frank CG Eyaid W Berger EG Aebi M Grubenmann CE Hennet T. Identification and functional analysis of a defect in the human ALG9 gene: definition of congenital disorder of glycosylation type IL. Am J Hum Genet. (2004) 75:146–50. 10.1086/422367 15148656 PMC1181998

[23] Chantret I Dancourt J Dupré T Delenda C Bucher S Vuillaumier-Barrot S A deficiency in dolichyl-P-glucose:Glc1Man9GlcNAc2-PP-dolichyl α3-glucosyltransferase defines a new subtype of congenital disorders of glycosylation. J Biol Chem. (2003) 278:9962–71. 10.1074/jbc.M211950200 12480927

[24] Hartung EA Guay-Woodford LM. Autosomal recessive polycystic kidney disease: a hepatorenal fibrocystic disorder with pleiotropic effects. Pediatrics. (2014) 134:e833–45. 10.1542/peds.2013-3646 25113295 PMC4143997

[25] Cadnapaphornchai MA Dell KM Gimpel C Guay-Woodford LM Gulati A Hartung EA Polycystic kidney disease in children: the current status and the next horizon. Am J Kidney Dis. (2025) 86:383–92. 10.1053/j.ajkd.2025.01.022 40113156 PMC12476570

[26] Stevens PE Ahmed SB Carrero JJ Foster B Francis A Hall RK KDIGO 2024 clinical practice guideline for the evaluation and management of chronic kidney disease. Kidney Int. (2024) 105:S117–314. 10.1016/j.kint.2023.10.018 38490803

[27] Gimpel C Fieuws S Hofstetter J Pitcher D Vanmeerbeek L Haeberle S Insights from ADPedKD, ERKReg and RaDaR registries provide a multi-national perspective on the presentation of childhood autosomal dominant polycystic kidney disease in high- and middle-income countries. Kidney Int. (2025) 108:105–18. 10.1016/j.kint.2025.02.026 40122340

[28] Burgmaier K Brinker L Erger F Beck BB Benz MR Bergmann C Refining genotype-phenotype correlations in 304 patients with autosomal recessive polycystic kidney disease and PKHD1 gene variants. Kidney Int. (2021) 100:650–9. 10.1016/j.kint.2021.04.019 33940108

[29] Gunay-Aygun M Tuchman M Font-Montgomery E Lukose L Edwards H Garcia A PKHD1 sequence variations in 78 children and adults with autosomal recessive polycystic kidney disease and congenital hepatic fibrosis. Mol Genet Metab. (2010) 99:160. 10.1016/j.ymgme.2009.10.010 19914852 PMC2818513

[30] Burgmaier K Kilian S Bammens B Benzing T Billing H Büscher A Clinical courses and complications of young adults with autosomal recessive polycystic kidney disease (ARPKD). Sci Rep. (2019) 9:7919. 10.1038/s41598-019-43488-w 31138820 PMC6538621

[31] MaBMRasoulyHMSabatelloM. To do or not to do—genetic testing for autosomal dominant polycystic kidney disease in children and adolescents. Kidney Int. (2025) 108:17–9. 10.1016/j.kint.2025.05.00140543935

